# Identifying Circulating MicroRNA in Kawasaki Disease by Next-Generation Sequencing Approach

**DOI:** 10.3390/cimb43020037

**Published:** 2021-06-25

**Authors:** Ken-Pen Weng, Ching-Feng Cheng, Kuang-Jen Chien, Luo-Ping Ger, Shih-Hui Huang, Kuo-Wang Tsai

**Affiliations:** 1 Congenital Structural Heart Disease Center, Department of Pediatrics, Kaohsiung Veterans General Hospital, Kaohsiung 813, Taiwan; kenpenweng@yahoo.com.tw (K.-P.W.); kjchien@vghks.gov.tw (K.-J.C.); 2 School of Medicine, National Yang Ming Chiao Tung University, Taipei 711, Taiwan; 3 Department of Physical Therapy, Shu-Zen Junior College of Medicine and Management, Kaohsiung 821, Taiwan; 4 Department of Pediatrics, Taipei Tzu Chi Hospital, Buddhist Tzu Chi Medical Foundation, Taipei 231, Taiwan; cfcheng@ibms.sinica.edu.tw; 5 Institute of Biomedical Sciences, Academia Sinica, Taipei 115, Taiwan; 6 Department of Pediatrics, Tzu Chi University, Hualien 970, Taiwan; 7 Department of Medical Education and Research, Kaohsiung Veterans General Hospital, Kaohsiung 813, Taiwan; lpger0329@gmail.com; 8 Department of Nursing, Fooyin University, Kaohsiung 831, Taiwan; sc042@fy.edu.tw; 9 Department of Research, Taipei Tzu Chi Hospital, Buddhist Tzu Chi Medical Foundation, Taipei 231, Taiwan

**Keywords:** microRNA, Kawasaki disease, circulating biomarker

## Abstract

Kawasaki disease (KD) typically occurs in children aged under 5 years and can cause coronary artery lesions (CALs). Early diagnosis and treatment with intravenous immunoglobulin can reduce the occurrence of CALs; therefore, identifying a good biomarker for diagnosing KD is essential. Here, using next-generation sequencing in patients with recurrent KD, those with viral infection, and healthy controls, we identified dysregulated circulating microRNAs as diagnostic biomarkers for KD. Pathway enrichment analysis illustrated the putative role of these miRNAs in KD progression. Their expression levels were validated using real-time polymerase chain reaction (qPCR). Fifteen dysregulated circulating miRNAs (fold changes >2 and <0.5) were differentially expressed in the recurrent KD group compared with the viral infection and control groups. These miRNAs were significantly involved in the transforming growth factor-β, epithelial–mesenchymal transition, and cell apoptosis signaling pathways. Notably, their expression levels were frequently restored after intravenous immunoglobulin treatment. Among the candidates, miR-24-3p expression level was significantly higher in patients with recurrent KD compared with healthy controls or viral infection controls (*p* < 0.001). Receiver operating characteristic analysis revealed that high miR-24-3p expression levels may be a potential biomarker for KD diagnosis. In conclusion, we identified miR-24-3p significantly higher in KD patients, which may be a potential diagnostic biomarker for KD.

## 1. Introduction

Kawasaki disease (KD) is a rare systemic inflammatory disease that typically occurs in children under 5 years of age [1]. KD is clinically diagnosed using several typical criteria, such as prolonged fever, oral mucosal changes, conjunctivitis, polymorphous rash, extremity changes, and lymphadenopathy [2]. Taiwan has the third-highest incidence of KD in the world, with 60 cases per 100,000 children [3,4,5]. Approximately 15–25% of children with KD are likely to develop coronary artery lesions (CALs); however, intravenous immunoglobulin (IVIG) treatment can reduce the frequency of CALs to 5% [2,3,6,7,8,9,10,11]. The etiology of KD remains unknown. It may be attributed to the combined effects of infection, immune response, and genetic susceptibility [1,12]. Delay in the diagnosis and treatment of KD can result in high CAL incidence, particularly in patients with atypical KD; accordingly, useful biomarkers for KD are required. Currently, KD diagnosis depends on clinical signs and inflammatory markers, including the erythrocyte sedimentation rate (ESR), C-reactive protein (CRP) level, and total leucocyte count. Except for these features and laboratory characteristics, cytokines are also used as auxiliary biomarkers for KD diagnosis, including interleukin (IL)-1, tumor necrosis factor-α, interferon-γ, IL-4, IL-6, IL-8, and IL-10 [13,14,15,16,17,18]. These cytokines have been shown to be significantly increased during acute KD. However, these laboratory data remain limited and nonspecific as diagnostic or prognostic biomarkers in KD. The availability of a favorable biomarker for early diagnosis of KD and treatment initiation with a single high dose of IVIG will help reduce the incidence of CALs [9,19].

MicroRNA (miRNA) is a class of small non-protein-coding RNAs that have critical regulatory functions in several physiological processes [20]. Large amounts of miRNAs exist in body fluids, including plasma [21,22,23,24,25,26]. These circulating miRNAs are derived from cell debris or actively transported by exosomes. Circulating miRNAs are stable and have reproducible properties, including resistance to RNase A digestion [25]. Therefore, they may serve as noninvasive biomarkers for KD diagnosis. Only a few studies have attempted to identify miRNA biomarkers for KD; and they have reported that dysregulation of circulating miR-93, miR-145, miR-200c, and miR-371-5p can act as a biomarker for KD diagnosis [27,28,29,30,31]. However, these results were obtained using a limited number of patients and a few specific miRNAs, with the biomarkers having insufficient sensitivity and specificity for KD diagnosis.

Although recurrent KD is extremely rare (approximately 0.8–3.5% of all KD cases), it is more severe than the initial episode [32,33,34]. Patients with recurrent KD are more likely to have an incomplete clinical presentation, thus at the risk of delaying diagnosis and IVIG treatment [35,36]. In the present study, we determined the miRNA profiles of plasma from patients with recurrent KD, healthy controls, and those with viral infection by using next-generation sequencing (NGS).

## 2. Materials and Methods

### 2.1. Clinical Samples and RNA Extraction

**Figure 1:**
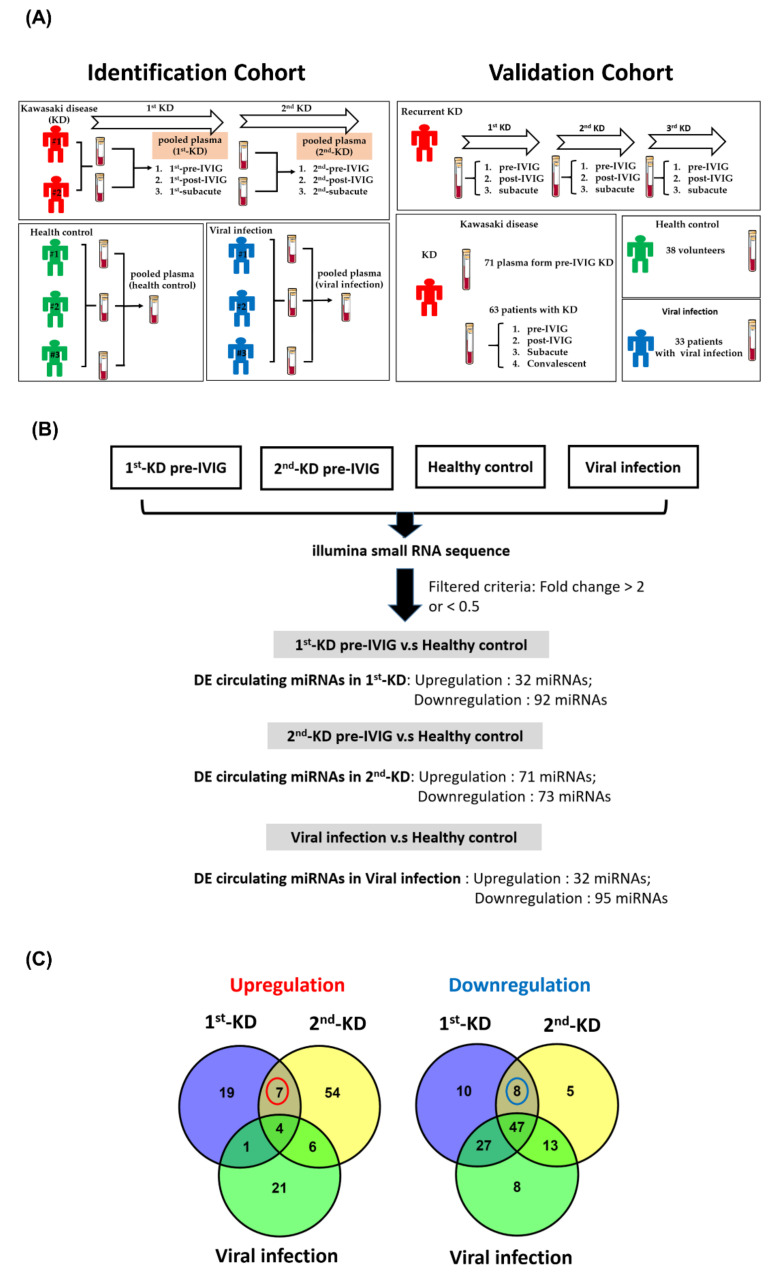
Identification of differentially expressed circulating microRNA (miRNA) in plasma by using next-generation sequencing (NGS). (**A**) In the identification cohort, a total of eight pooled plasmas were prepared for small RNA profiling by next-generation sequencing approach (left panel). For the validation cohort, we recruited 143 plasma samples (38 healthy controls, 33 participants with viral infection, and 72 patients with KD). (**B**) Flowchart of identification of differentially expressed (DE) circulating miRNAs in plasma by obtaining four profiles through NGS. The DE circulating miRNAs were identified in 1st KD-pre-IVIG, 2nd KD-pre-IVIG, and viral infection by comparing with healthy control. Furthermore, the numbers of DE circulating miRNAs 1st KD-pre-IVIG, 2nd KD-pre-IVIG, and viral infection are shown, respectively. (**C**) Venn diagrams of the number of upregulated and downregulated circulating miRNAs in patients with recurrent KD.

This study was performed at the Department of Pediatrics, Kaohsiung Veterans General Hospital (KVGH), Taiwan. Medical records of all children who fulfilled the diagnostic criteria for KD in our hospital between 1997 and 2019 were reviewed. Exclusion criteria included missing clinical data, no IVIG treatment, or initial IVIG treatment beyond 10 days of fever. A total of 74 KD patients (age: 24.5 ± 22.1 months, Male/Female ratio: 1.85) were collected, including 3 patients with recurrent KD. Forty-one healthy controls (age: 30.2 ± 16.8 months, Male/Female ratio: 1.3) without cardiovascular disease and a history of KD were recruited. The other control group consisted of 36 patients with viral infection (age: 26.7 ± 15.8 months, Male/Female ratio: 1.57) as well as without a history of KD. Using these clinical samples, we identified circulating miRNAs in the identification cohort by the next-generation sequencing approach and confirmed their expression in the validation cohort by real-time PCR. The first pooled plasma sample was collected from two patients suffering from KD for the first time, including three stages, pre-IVIG treatment, post-IVIG treatment, and subacute stage. The second plasma sample was obtained from identical patients who had recurrent KD. The pooled samples in the period of recurrent KD were also collected at different stages, including pre-IVIG treatment, post-IVIG treatment, and subacute stage. In addition, the pooled plasma of three healthy volunteers and three patients with viral infection (fever) was obtained from controls (Figure 1A, left panel). Selected miRNAs for KD diagnosis were further validated using real-time polymerase chain reaction (PCR). For the validation cohort, we recruited 143 people (38 healthy controls, 33 participants with viral infection, and 72 patients with KD). Among the patients, one patient suffered from KD three times (Figure 1A, right panel). In addition, plasma was collected from the 63 patients with KD at four stages: pre-IVIG treatment, post-IVIG treatment, subacute, and convalescent. The study protocol was approved by the Institutional Review Board of KSVGH (VGHKS10-CT9-04), and all patients provided informed consent. Plasma small RNA was extracted from 200 μL of plasma by using the miRNeasy Serum/Plasma Kit (Qiagen, Valencia, CA, USA) according to the manufacturer’s instructions. Finally, RNA was resuspended in 14 μL of RNase-free H_2_O.

### 2.2. Generation of Small RNA Profiles through NGS

Eight plasma samples were prepared by pooling equal amounts of plasma from two patients with recurrent KD at different stages (1st-pre-IVIG treatment, 1st-post-IVIG treatment, 1st-subacute, 2nd-pre-IVIG treatment, 2nd-post-IVIG treatment, and 2nd-subacute), three participants with viral infection (Fever control), and three healthy participants (Healthy control), respectively (Figure 1A, left panel). A total of 300 μL of pooled plasma was used for RNA extraction, and small RNA libraries were constructed with the Small RNA Library Preparation Kit (Illumina, San Diego, CA, USA). Next, small RNA sequences were generated using the Illumina HiSeq platform (Illumina, San Diego, CA, USA). Details are presented in our previous study [37]. Finally, these sequence reads were subjected to quality control, and low-quality reads were removed in accordance with the standard small RNA analysis pipeline [38]. The quality of sequence reads was measured using a metric called Q score. The Q score of 30 was considered acceptable [39].

### 2.3. Mapping Clean Reads to Pre-miRNAs

To investigate miRNA expression profiles in eight libraries (pre-IVIG treatment, post-IVIG treatment, and subacute stages of the first and second KD episodes; healthy control; and viral infection control), we mapped the qualified clean reads back to human pre-miRNAs (miRbase-19) by using the bowtie tool [40]. Finally, the expression levels of individual miRNAs were calculated by using our previously developed miRSeq tool [41]. The expression levels of individual miRNAs in each library are presented as transcripts per million.

### 2.4. Identifying Differentially Expressed miRNAs and Pathway Enrichment Analysis

Circulating miRNAs with differential expression were filtered using the following thresholds: transcripts per million (TPM) (first Kawasaki disease (KD) (1st-KD) + recurrent KD (2nd-KD) + healthy control + viral infection) > 10 and fold change ≥ 2 or fold change < 0.5. A total of 15 differentially expressed miRNAs (including seven upregulated miRNAs and eight downregulated miRNAs) were identified in the KD group compared with the viral infection control group. We further identified their putative targets by using the TargetScan tool [42]. These putative targets of the seven upregulated and eight downregulated circulating miRNAs were subjected to pathway enrichment analysis by using the MetaCore software (GeneGo, St. Joseph, MI, USA).

### 2.5. TaqMan Real-Time PCR Assay

After RNA extraction, the total RNA (2 μL) was used for reverse transcription assay with a TaqMan Advanced cDNA Synthesis kit (Applied Biosystems, Foster City, CA, USA). Briefly, 2 μL of total RNA was polyadenylated at 37 °C for 45 min, with the polyadenylation then stopped at 65 °C for 10 min. The polyadenylated small RNAs were ligated with an adapter at 16 °C for 60 min. After ligation, complementary DNA (cDNA) was synthesized through reverse transcription reaction with a universal reverse primer at 42 °C for 15 min. Finally, cDNA was further amplified as follows: 95 °C for 20 s, followed by 14 cycles of 95 °C/3 s and 60 °C/30 s, with a final stop at 99 °C for 10 min by using a PCR thermocycler. The expression levels of mature miR-24-3p (Catalog number: 000402), miR-99b-5p (Catalog number: 000436), miR-30e-5p (Catalog number: 000422), miR-128-3p (Catalog number: 002216), and miR-16 (Catalog number: 000391) were examined using TaqMan miRNA assay kits (Applied Biosystems, Foster City, CA, USA). The miR-16 was used for internal control [43], and the expression levels of individual miRNA were calculated and quantified through △C_T_ (△C_T_ = C_TmiRNA candidates_ − C_TmiR-16_) [44].

### 2.6. Statistical Analysis

The expression levels of miRNAs in patients with KD, healthy controls, and viral infection controls obtained from TaqMan real-time PCR were analyzed using the Student’s *t*-test. The difference was considered significant at *p* < 0.05. The diagnostic value of miRNA as a biomarker for KD diagnosis was evaluated using the receiver operating characteristic (ROC) curves. Based on the optimal cutoff value of selected miRNAs, a ROC curve was generated, and the area under the curve (AUC) was calculated to evaluate the specificity and sensitivity for differentiating KD patients from healthy controls or those with viral infection. Detailed information was described in our previous study [26]. *p* < 0.05 was considered statistically significant.

## 3. Results

### 3.1. Generating miRNA Profiles of Plasma from Recurrent KD and Control Groups

To identify circulating miRNAs as biomarkers for KD, we generated eight miRNA expression profiles: those from patients with KD (pre-IVIG treatment, post-IVIG treatment, subacute stages of patients suffering from KD first time, and those with recurrent.), patients with viral infection, and healthy controls. Total RNA of the eight pooled plasma samples was extracted, and small RNA sequencing was performed using NGS. As presented in Table 1, we obtained more than 6 million clean reads in eight libraries. After mapping the clean reads to the miRNA database, approximately 100 miRNAs expressed in the plasma were detected in our samples. A considerable amount of small RNAs in the plasma from the eight clinical samples were 21–23 nt in length, which is consistent with the ideal size of miRNAs (Supplementary Figure S1).

### 3.2. Circulating miRNAs in Recurrent KD

**Figure 2:**
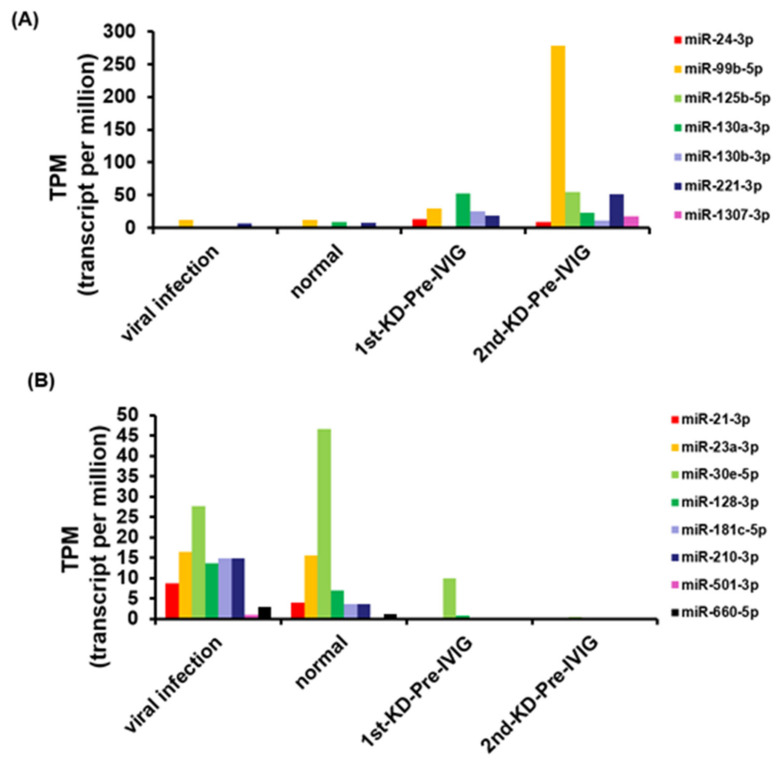
Expression levels of 15 miRNA candidates, obtained by analyzing NGS data. (**A**) The expression levels of seven circulating miRNAs were higher in the recurrent KD group than in the healthy and viral infection control groups. (**B**) The expression levels of eight circulating miRNAs were decreased in the recurrent KD group compared with the healthy and viral infection control groups.

In order to identify circulating miRNAs for KD diagnosis, we performed small RNA profiles of eight pooled plasma samples using the NGS approach (Figure 1A). A good KD diagnostic marker can not only distinguish the healthy control group but also effectively distinguish it from the viral infection group. Therefore, we used a series of selecting steps to identify recurrent KD-specific circulating miRNAs. Figure 1B presents a detailed flowchart showing how DE circulating miRNA candidates were selected from the small RNA profiles of the patients with recurrent KD, healthy controls, and viral controls. First, we discovered that 31 and 92 miRNAs were upregulated and downregulated, respectively, with a two-fold change in patients suffering from KD first time (1st-pre-IVIG) compared with the healthy controls, respectively (Figure 1B and Supplementary Table S1). Furthermore, we identified 71 and 73 miRNAs that were upregulated and downregulated, respectively, in the plasma of the recurrent KD episode (2nd-pre-IVIG) compared with those of the healthy controls (Figure 1B and Supplementary Table S2). Among these miRNAs, 11 and 55 miRNAs were consistently upregulated and downregulated, respectively, in the plasma from both the 1st-pre-IVIG and 2nd-pre-IVIG compared with the healthy control. However, these differentially expressed miRNAs might have resulted from viral infection-induced. In addition, 32 and 95 miRNAs were upregulated and downregulated in the viral infection group compared with those of the healthy control group, respectively (Figure 1B and Supplementary Table S3). In order to identify recurrent-KD-specific induced circulating miRNAs, we further subtracted the fever-induced miRNAs. As shown in Figure 1C, our results revealed that only a small fraction of miRNAs exhibited differential expression (> or < two-fold change) in patients with recurrent KD compared with the viral infection control. The results when the viral infection-induced or KD-induced DE circulating miRNAs were compared revealed that seven miRNAs (miR-24-3p, miR-99b-5p, miR-125b-5p, miR-130a-3p, miR-130b-3p, miR-221-3p, and miR-1307-3p) and eight miRNAs (miR-21-3p, miR-23a-3p, miR-30e-5p, miR-128-3p, miR-181c-5p, miR-210-3p, miR-501-3p, and miR-660-5p) were upregulated and downregulated specifically in the plasma of patients with recurrent KD, respectively (Figure 2A,B).

We evaluated the putative signaling pathway comodulated by seven upregulated and eight downregulated miRNAs and predicted their putative target genes by using the bioinformatics approach. We then examined these genes using MetaCore pathway analytical tools. A total of 127 target genes were predicted to be targets of miR-24-3p, miR-99b-5p, miR-125b-5p, miR-130a-3p, miR-130b-3p, miR-221-3p, and miR-1307-3p, and these targets were revealed to be significantly enriched in AKT signaling, apoptosis-related signaling, cytoskeleton remodeling, the cell cycle, immune response, and transforming growth factor (TGF)-β signaling (Table 2). In addition, 192 target genes were predicted to be targets of miR-21-3p, miR-23a-3p, miR-30e-5p, miR-128-3p, miR-181c-5p, miR-210-3p, miR-501-3p, and miR-660-5p. These genes were found to be significantly involved in epithelial–mesenchymal transition (EMT), TGF-β signaling, cytoskeleton remodeling, immune response, and apoptosis-related signaling (Table 3). Taken together, our data revealed that the differentially expressed miRNAs played roles in modulating TGF-β signaling, cell apoptosis, EMT, and the immune response signaling pathway.

### 3.3. Circulating MiRNAs during KD Progression

**Figure 3:**
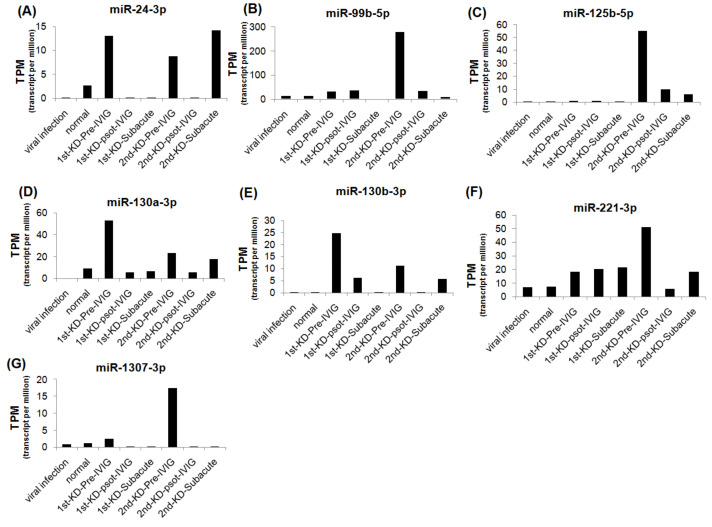
Expression levels of seven upregulated miRNA candidates in recurrent KD at different stages. The expression levels of miR-24-3p (**A**), miR-99b-5p (**B**), miR-125b-5p (**C**), miR-130a-3p (**D**), miR-130b-3p (**E**), miR-221-3p (**F**), and miR-1307-3p (**G**) were analyzed using NGS; miRNA expression levels are presented as TPM.

**Figure 4:**
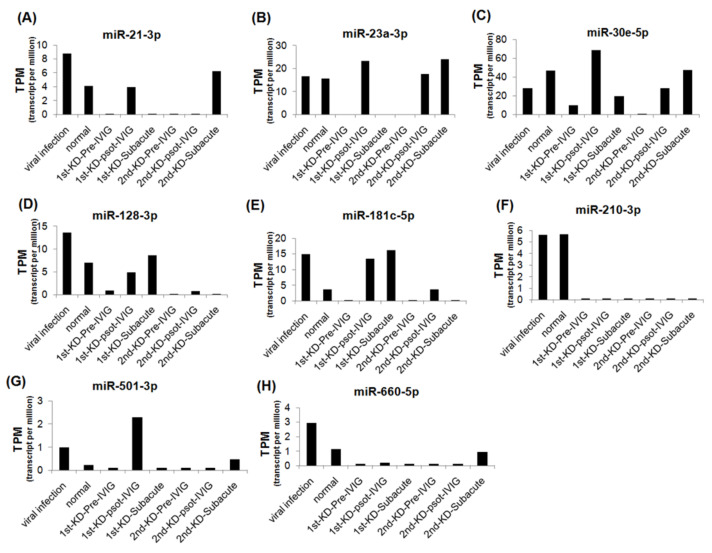
Expression levels of eight downregulated miRNAs in recurrent KD at different stages. The expression levels of miR-21-3p (**A**), miR-23a-3p (**B**), miR-30e-5p (**C**), miR-128-3p (**D**), miR-181c-5p (**E**), miR-210-3p (**F**), miR-501-3p (**G**), and miR-660-5p (**H**) were analyzed using NGS; miRNA expression levels are presented as TPM.

**Figure 5:**
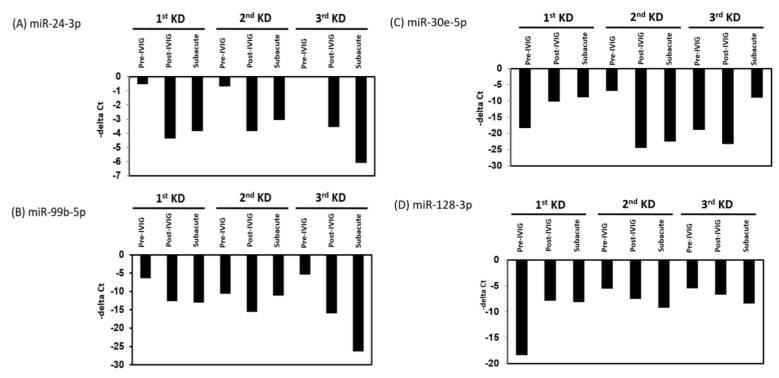
Expression levels of miR-24-3p, miR-99b-5p, miR-30e-5p, and miR-128-3p in patients with recurrent KD. The relative levels of miR-24-3p (**A**), miR-99b-5p (**B**), miR-30e-5p (**C**), and miR-128-3p (**D**) were examined in plasma from patients with KD, and those relapsed with second and third times at different stages using the TaqMan real-time polymerase chain reaction (PCR). miR-16 was used as an internal control to normalize the expression levels of miRNA candidates (deltaCt_miRNA candidates_ = Ct_miRNA candidates_ − Ct_miR-16_).

We further analyzed the expression levels of seven upregulated and eight downregulated miRNA candidates in patients with KD at different stages. The expression levels of the upregulated miRNAs were decreased, and those of the downregulated miRNAs were increased after IVIG treatment and in the subacute stage (Figure 3 and Figure 4). We examined the expression levels of miR-24-3p, miR-99b-5p, miR-30e-5p, and miR128-3p by using real-time PCR in a very rare patient with KD who suffered from three recurrences (Figure 1A, right panel). The miR-24-3p and miR-99b-5p expression levels gradually decreased with recurrent KD after IVIG treatment (Figure 5A,B). Furthermore, the miR-30e-5p and miR-128-3p expression levels were increased in patients with KD for the first time after the post-IVIG treatment and subacute stages, but the levels were decreased in the recurrent KD episodes after IVIG treatment (Figure 5C,D).

### 3.4. Circulating MiR-24-3p Levels in Patients with KD

**Figure 6:**
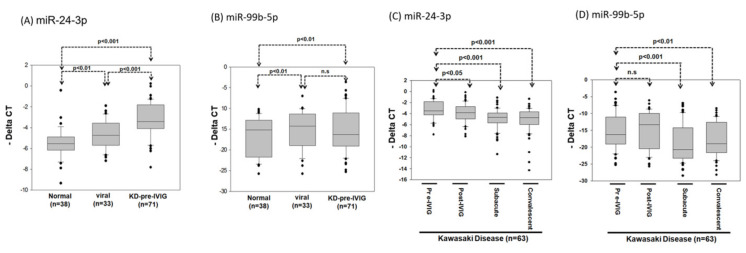
The expression levels of miR-24-3p and miR-99b-5p in the plasma of patients with KD and viral infection or healthy control. The relative expression levels of miR-24-3p (**A**) and miR-99b-5p (**B**) were examined in plasma from 38 healthy controls, 33 participants with viral infection, and 71 patients with KD using the TaqMan real-time PCR. (**C**,**D**) The relative expression levels of miR-24-3p and miR-99b-5p were examined in plasma from 63 patients with KD at four stages: pre-IVIG treatment, post-IVIG treatment, subacute, and convalescent using the TaqMan real-time PCR. The miR-16 was used as an internal control to normalize the expression levels of miRNA candidates (deltaCt_miRNA candidates_ = Ct_miRNA candidates_ − Ct_miR-16_).

In general, high-expression miRNA is more suitable as a diagnostic biomarker. Therefore, we further evaluated whether miR-24-3p and miR-99b-5p expression levels can serve as biomarkers for KD diagnosis. We collected 142 additional plasma samples: from 38 healthy controls, 33 patients with viral infection, and 71 patients with KD. After RNA extraction, cDNA conversion was performed using a TaqMan Advanced cDNA Synthesis kit. The miR-24-3p and miR-99b-5p expression levels were examined using real-time PCR. As presented in Figure 6A, the miR-24-3p expression levels were significantly higher in the viral infection and KD groups than in the healthy control group (*p* < 0.01 and 0.001, respectively) and were significantly higher in the KD group than the viral infection group (*p* < 0.001; Figure 6A). In addition, the miR-99b-5p expression levels were significantly higher in the viral infection and KD groups than the healthy control group (*p* < 0.01), whereas no difference was observed between the viral infection and KD groups (Figure 6B).

**Figure 7:**
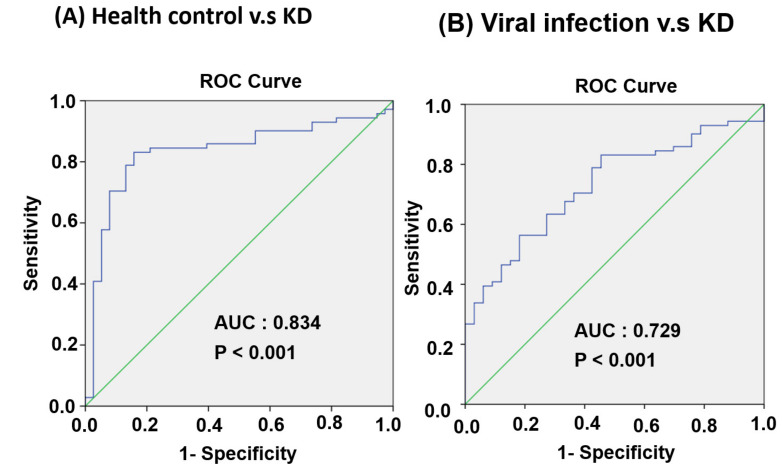
Evaluation of plasma miR-24-3p for the diagnosis of patients with KD. ROC curves comparing plasma miR-24-3p expression levels between the KD and control groups. (**A**) Healthy control and KD groups. (**B**) Viral infection and KD groups.

We examined the miR-24-3p and miR-99b-5p expression levels in 63 patients with KD at four different stages: the pre-IVIG, post-IVIG, subacute, and convalescent stages. As illustrated in Figure 6C, miR-24-3p expression gradually decreased at the post-IVIG (*p* < 0.05), subacute (*p* < 0.001), and convalescent stages (*p* < 0.001) after patients received IVIG; notably, its expression levels were similar to those in healthy controls. However, miR-99b-5p expression was significantly lower in the subacute (*p* < 0.001) and convalescent stages (*p* < 0.01) than in the pre-IVIG stage (Figure 6D). Taken together, these data implied that miR-24-3p may be a biomarker for KD diagnosis. ROC curve analysis was performed to evaluate its diagnostic accuracy (Figure 7A,B). The miR-24-3p expression levels were significantly higher in the patients with KD than in the healthy controls (Figure 6A), with the area under the ROC curve being 0.834 (95% confidence interval [CI] 0.751–0.916; *p* < 0.001). At the optimal miR-24-3p expression cutoff of −4.56, the sensitivity and specificity were 83.1% and 84.2%, respectively. Furthermore, the miR-24-3p expression levels in the patients with KD were significantly higher than those in the viral infection group (Figure 6B), with the area under the ROC curve being 0.729 (95% CI 0.631–0.826; *p* < 0.001). At the optimal miR-24-3p expression cutoff of −3.53, the sensitivity and specificity were 56.3% and 81.8%, respectively.

## 4. Discussion

In this study, we identified seven upregulated and eight downregulated circulating miRNAs in patients with KD compared with the levels in patients with viral infection, and the altered expression levels of these miRNAs were reversed after IVIG treatment. Another study also reported that decreased miR-155 and miR-21 expression levels were restored after IVIG treatment [28]. IVIG is usually administered as an anti-inflammatory and immunomodulatory agent in autoimmune diseases. The detailed mechanism of IVIG treatment in patients with KD is unclear. In the present study, pathway enrichment analysis revealed that the dysregulated miRNA candidates were significantly involved in the TGF-β signaling pathway and EMT (Table 2 and Table 3). TGF-β may contribute to aneurysm formation by inducing myofibroblast generation through the EMT in the arterial wall of patients with KD [45]. TGF-β expression levels were shown to increase after IVIG treatment, indicating that it is also critical in the anti-inflammatory response [46]. Our data revealed that miR-24-3p expression levels were significantly higher in the KD group compared with the healthy and viral infection control groups and that miR-24-3p expression levels significantly decreased after IVIG treatment. Accumulating evidence has revealed that miR-24 regulates cardiac fibrosis by modulating the furin–TGF-β pathway [47,48,49]. Together, these findings imply that miR-24-3p plays a critical role in KD progression through the TGF-β signaling pathway. However, further research is required to elucidate the mechanism of miR-24-3p involved in KD with IVIG treatment.

Delayed diagnosis and treatment of KD can result in a high incidence of CALs, particularly in patients with atypical KD; therefore, useful biomarkers for KD are required. Several putative miRNA candidates have been identified [27,28,29,30,31]. Shimizu et al. demonstrated that miR-145 and miR-145 * may regulate TGF-β pathway-associated gene expression during the acute stage of KD [29]. Yun et al. reported that miR-200 and miR-371 expression levels were significantly higher in patients with acute KD than in controls and that these miRNAs were involved in the inflammatory response and a crucial mechanism for KD pathogenesis [27]. Ni et al. revealed that FoxP3+ Treg expression levels were low in patients with acute KD, resulting from the silencing of miR-155-associated signaling and upregulation of miR-31 signaling [28]. Taken together, these few studies revealed that miRNAs can be used as noninvasive biomarkers to diagnose KD or predict KD progression. Recently, Kuo et al. identified eight dysregulated miRNAs (miR-27a-3p, miR-30c-5p, miR-30e-3p, miR-148a-3p, miR-182-5p, miR-183-5p, miR-378a-3p, and miR-941) in whole-blood samples of patients with KD and compared them with those of patients with fever [50]. Using a support vector machine model, they developed KD biomarker panels for KD diagnosis on the basis of these dysregulated miRNA expression levels [50]. In the present study, we used plasma samples and identified different miRNA candidates from those reported by Kuo et al. Our data revealed that the miR-24-3p expression level is a potential biomarker for KD diagnosis.

Some limitations in this study need to be specified. This is a single-center investigation with a limited number of patients. Serum proteins related to the identified miRNAs were not measured. A multi-center study with a large cohort is suggested.

In conclusion, we identified miR-24-3p significantly higher in KD patients, which may be a potential diagnostic biomarker for KD.

## Figures and Tables

**Table 1:** Categories of sequence reads in the eight libraries.

Sample Name	Total Illumina Reads	# Clean (% Percentage)	Detected miRNAs#
Viral infection	6,689,522	6,444,268 (98.03%)	117
Healthy control	7,616,599	7,460,596 (98.88%)	155
1st KD-pre-IVIG	7,945,909	7,442,444 (94.6%)	123
1st KD-post-IVIG	7,881,043	7,658,251 (98.15%)	137
1st KD-subacute	8,056,044	7,841,016 (98.23%)	127
2nd KD-pre-IVIG	6,912,925	6,468,229 (94.67%)	123
2nd KD-post-IVIG	6,734,488	6,457,836 (97.034%)	146
2nd KD-subacute	7,230,145	7,032,058 (98.19%)	162

**Table 2:** Targets of upregulated circulating microRNAs in the plasma of patients with Kawasaki disease (KD) were involved in canonical pathway maps.

Pathway Maps	Total	*p*-Value	FDR	Genes from Active Data
1. Signal transduction_AKT signaling	43	3.540 × 10^−12^	1.349 × 10^−9^	p21, Bim, FOXO3A, PTEN, mTOR, p27KIP1, c-Myc, MDM2, PI3K reg class IA, HGF receptor (Met)
2. Apoptosis and survival_p53-dependent apoptosis	29	1.289 × 10^−10^	2.455 × 10^−8^	Bim, Apaf-1, Bcl-2, MEK4(MAP2K4), p38alpha (MAPK14), BMF, MDM2, p14ARF
3. Apoptosis and survival_Cytoplasmic/mitochondrial transport of proapoptotic proteins Bid, Bmf and Bim	34	5.254 × 10^−10^	6.672 × 10^−8^	TRAF2, Bim, Apaf-1, Bcl-2, MEK4(MAP2K4), BMF, MKK7 (MAP2K7), TNF-alpha
4. Cytoskeleton remodeling_TGF, WNT and cytoskeletal remodeling	111	4.303 × 10^−9^	4.099 × 10^−7^	p21, p38 MAPK, FOXO3A, mTOR, ROCK, NLK, c-Myc, MDM2, PI3K reg class IA, Dsh, TGF-beta receptor type II
5. Cell cycle_Regulation of G1/S transition (part 1)	38	4.209 × 10^−8^	3.207 × 10^−6^	p21, CDC25A, p27KIP1, SMAD4, TGF-beta receptor type II, CDK6, p16INK4
6. Translation_Non-genomic (rapid) action of Androgen Receptor	40	6.127 × 10^−8^	3.891 × 10^−6^	ErbB2, FOXO3A, PTEN, mTOR, PI3K reg class IA (p85-alpha), MDM2, Dsh
7. Immune response_IL-15 signaling	64	1.027 × 10^−7^	5.589 × 10^−6^	p38 MAPK, TRAF2, Bcl-2, mTOR, MEK4(MAP2K4), c-Myc, ETS1, PI3K reg class IA (p85)
8. Apoptosis and survival_Regulation of Apoptosis by Mitochondrial Proteins	33	4.490 × 10^−7^	1.595 × 10^−5^	Bak, Bim, Apaf-1, Bcl-2, BMF, PUMA
9. Cell cycle_ESR1 regulation of G1/S transition	33	4.490 × 10^−7^	1.595 × 10^−5^	p21, ESR1 (nuclear), CDC25A, p27KIP1, c-Myc, CDK6
10. Apoptosis and survival_Endoplasmic reticulum stress response pathway	53	4.604 × 10^−7^	1.595 × 10^−5^	Bak, TRAF2, Bim, Apaf-1, Bcl-2, MEK4(MAP2K4), p38alpha (MAPK14)

**Table 3:** Targets of downregulated circulating microRNAs in the plasma of patients with KD were involved in canonical pathway maps.

Pathway Maps	Total	*p*-Value	FDR	Genes from Active Data
1. Development_Regulation of epithelial-to-mesenchymal transition (EMT)	64	9.407 × 10^−13^	3.612 × 10^−10^	SNAIL1, IL-1 beta, SMAD2, NOTCH4, Jagged1, Bcl-2, TGF-beta 1, SIP1 (ZFHX1B), WNT, SP1, TNF-alpha, TGF-beta receptor type II, Tropomyosin-1
2. Development_TGF-beta-dependent induction of EMT via SMADs	35	1.120 × 10^−11^	2.150 × 10^−9^	HMGA2, SNAIL1, SMAD2, SMAD4, Jagged1, TGF-beta 1, SIP1 (ZFHX1B), TGF-beta, SP1, TGF-beta receptor type II
3. Development_TGF-beta receptor signaling	50	5.340 × 10^−10^	6.835 × 10^−8^	Ski, XIAP, SMAD2, NFKBIA, SMAD4, MEK3(MAP2K3), TGF-beta 1, SMAD7, SP1, TGF-beta receptor type II
4. Cytoskeleton remodeling_TGF, WNT and cytoskeletal remodeling	111	1.306 × 10^−9^	1.254 × 10^−7^	NLK, XIAP, SMAD2, PLAT (TPA), MEK3(MAP2K3), DOCK1, FOXO3A, Cofilin, TGF-beta 1, WNT, SP1, TGF-beta receptor type II, Collagen IV
5. Cell adhesion_Plasmin signaling	35	1.003 × 10^−8^	7.701 × 10^−7^	XIAP, PLAT (TPA), TGF-beta R III (betaglycan), MEK3(MAP2K3), TGF-beta 1, Neuroserpin, TGF-beta receptor type II, Collagen IV
6. Immune response_HMGB1/RAGE signaling pathway	53	1.873 × 10^−8^	1.199 × 10^−6^	K-RAS, ICAM1, IL-1 beta, NFKBIA, PLAT (TPA), I-kB, MEF2C, SP1, TNF-alpha
7. Cytoskeleton remodeling_Cytoskeleton remodeling	102	6.356 × 10^−7^	3.487 × 10^−5^	PTEN, XIAP, PLAT (TPA), MEK3(MAP2K3), DOCK1, Cofilin, TGF-beta 1, MyHC, TGF-beta receptor type II, Collagen IV
8. Possible pathway of TGF-beta 1-dependent inhibition of CFTR expression	27	9.252 × 10^−7^	4.441 × 10^−5^	XIAP, SMAD4, MEK3(MAP2K3), TGF-beta 1, SMAD7, TGF-beta receptor type II
9. Apoptosis and survival_FAS signaling cascades	44	1.225 × 10^−6^	5.225 × 10^−5^	Bim, Lamin B, XIAP, FasL(TNFSF6), Apaf-1, Bcl-2, DAXX
10. Development_BMP signaling	33	3.252 × 10^−6^	1.249 × 10^−4^	Ski, XIAP, SMAD4, MEK3(MAP2K3), BMP receptor 2, SMAD7

## Data Availability

The data that support the findings of this study are available from the corresponding author upon reasonable request.

## Supplementary Materials

The following are available online at https://www.mdpi.com/article/10.3390/cimb43020037/s1, Figure S1: The distribution of small RNAs of various lengths (18–30 bp) sequenced by next-generation sequencing, Table S1: The differential expressed miRNAs in patients suffering with KD first time (1st-KD -pre-IVIG) compared with health control, Table S2: The differential expressed miRNAs in recurrent KD (2ndKD-pre-IVIG) compared with healthyl control, Table S3: The differentially expressed miRNAs in patients with viral infection compared with healthy control.
