# Supplementary material for: Identifying Circulating MicroRNA in Kawasaki Disease by Next-Generation Sequencing Approach
Source: Curr Issues Mol Biol. 2021 Jun 25;43(2):485–500. doi: 10.3390/cimb43020037 (PMC8929010; doi:10.3390/cimb43020037)
Supplement: Supplementary file 1 [file cimb-43-00037-s001.zip › cimb-1227565-supplementary.pdf]

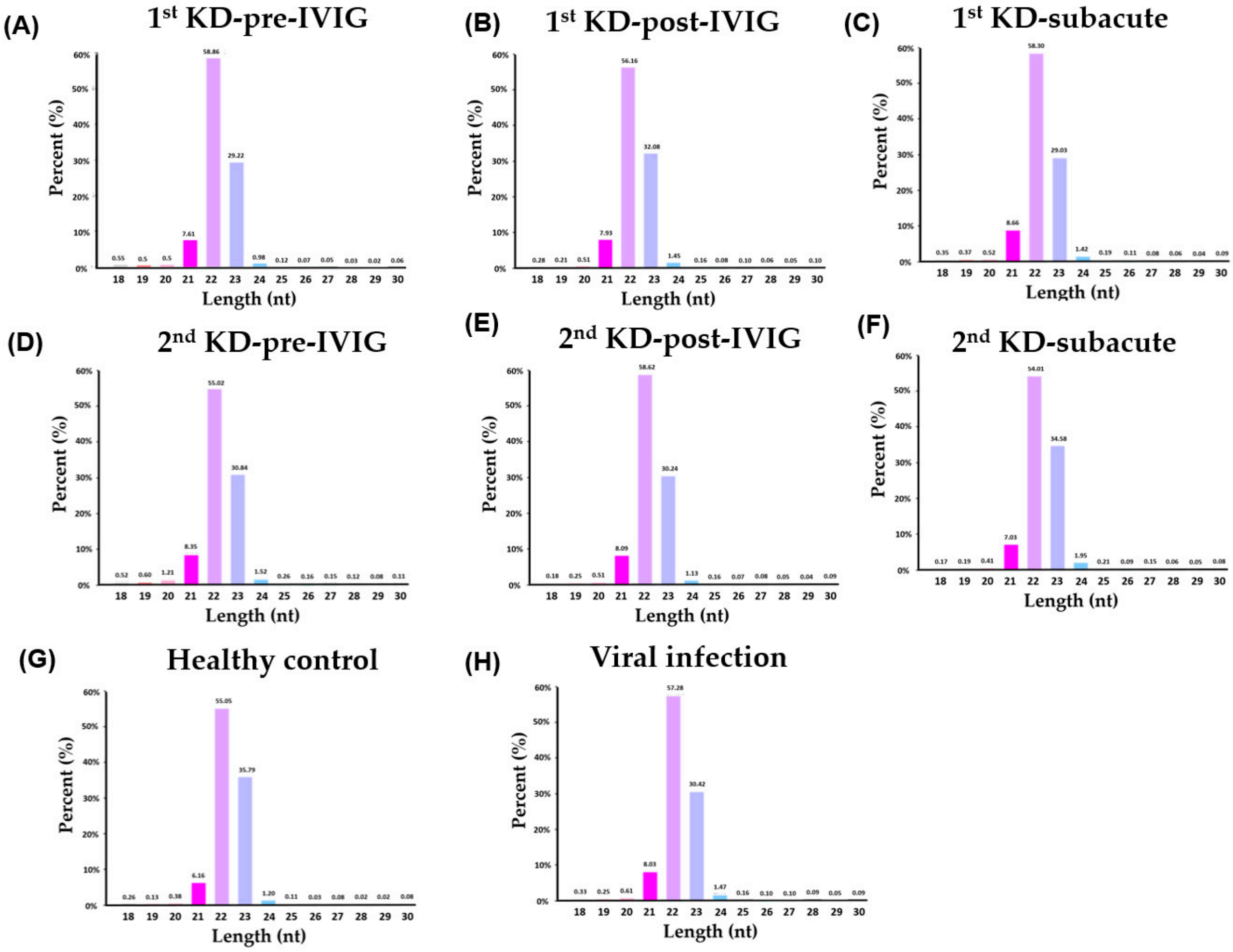

Supplementary Figure S1. The distribution of small RNAs of various lengths (18–30 bp) sequenced by next-generation sequencing. Next-generation sequencing analysis of circulating microRNAs from two pooled plasma with Kawaskai disease (KD) of the first and recurrent KD episodes at different stages of the disease. (A) 1st KD-pre- IVIG, (B) 1st KD-post-IVIG, (C) 1st KD-subacute stages, (D) 2nd KD-pre- IVIG, (E) 2nd KD-post-IVIG, (F) 2nd KD-subacute, (G) healthy control and (H) viral infection.

Supplementary Table S1. The differential expressed miRNAs in patients suffering with KD first time (1st-KD -pre-IVIG) compared with health control

microRNA upregulation	Fold change	microRNA downregulation	Fold change
hsa-miR-130b-3p	247.3	hsa-miR-19a-3p	0.002
hsa-miR-30b-5p	140.1	hsa-miR-4732-3p	0.005
hsa-miR-99a-5p	98.9	hsa-miR-23a-3p	0.006
hsa-let-7d-3p	92.7	hsa-miR-30d-5p	0.007
hsa-miR-148b-3p	80.4	hsa-miR-223-3p	0.008
hsa-miR-584-5p	72.1	hsa-miR-145-5p	0.009
hsa-miR-1273h-3p	66.0	hsa-miR-340-5p	0.009
hsa-miR-3158-3p	55.6	hsa-miR-183-5p	0.009
hsa-miR-425-3p	51.5	hsa-miR-30e-3p	0.010
hsa-miR-151b	47.4	hsa-miR-6511a-3p	0.012
hsa-miR-3613-5p	45.3	hsa-miR-144-5p	0.013
hsa-miR-197-3p	39.2	hsa-miR-210-3p	0.018
hsa-miR-100-5p	38.2	hsa-miR-625-3p	0.019
hsa-miR-744-5p	6.2	hsa-miR-15b-5p	0.021
hsa-miR-130a-3p	5.7	hsa-miR-17-5p	0.023
hsa-miR-24-3p	4.8	hsa-miR-21-3p	0.025
hsa-miR-615-3p	4.1	hsa-miR-598-3p	0.026
hsa-miR-138-5p	4.1	hsa-miR-18a-3p	0.026
hsa-miR-185-5p	3.9	hsa-miR-664b-3p	0.028
hsa-miR-4732-5p	3.8	hsa-miR-93-3p	0.030
hsa-let-7d-5p	3.4	hsa-miR-199a-3p	0.032
hsa-miR-652-3p	3.4	hsa-miR-199b-3p	0.032
hsa-miR-125b-5p	2.7	hsa-miR-28-3p	0.032
hsa-miR-221-3p	2.5	hsa-miR-139-5p	0.032
hsa-miR-99b-5p	2.5	hsa-miR-486-3p	0.032
hsa-miR-146a-5p	2.2	hsa-miR-20a-5p	0.034
hsa-miR-1307-3p	2.2	hsa-miR-339-5p	0.034
hsa-miR-423-3p	2.1	hsa-miR-4742-3p	0.034
hsa-miR-132-3p	2.1	hsa-miR-194-5p	0.034
hsa-miR-409-5p	2.1	hsa-miR-378a-3p	0.034
hsa-miR-411-5p	2.1	hsa-miR-335-5p	0.037
		hsa-miR-589-5p	0.037
		hsa-miR-3200-5p	0.037
		hsa-miR-424-3p	0.037
		hsa-miR-425-5p	0.037
		hsa-miR-301a-3p	0.040
		hsa-miR-4446-3p	0.040
		hsa-miR-6754-3p	0.040
		hsa-miR-30c-5p	0.040
		hsa-miR-125b-2-3p	0.040
		hsa-miR-483-3p	0.049
		hsa-miR-339-3p	0.055
		hsa-miR-32-5p	0.055
		hsa-miR-636	0.055
		hsa-miR-125a-5p	0.057
		hsa-miR-181c-5p	0.057
		hsa-miR-106a-5p	0.063
		hsa-miR-548ad-5p	0.063
		hsa-miR-548ae-5p	0.063
		hsa-miR-548ay-5p	0.063
		hsa-miR-548d-5p	0.063
		hsa-miR-1180-3p	0.063
		hsa-miR-19b-3p	0.066
		hsa-miR-181a-2-3p	0.068
		hsa-miR-106b-3p	0.072
		hsa-miR-204-5p	0.083
		hsa-miR-22-5p	0.089
		hsa-miR-660-5p	0.089
		hsa-miR-150-3p	0.089
		hsa-let-7e-5p	0.102
		hsa-miR-128-3p	0.118
		hsa-miR-150-5p	0.123
		hsa-miR-26a-5p	0.149
		hsa-miR-30e-5p	0.212
		hsa-miR-92b-3p	0.213
		hsa-miR-146b-5p	0.222
		hsa-miR-182-5p	0.232
		hsa-miR-98-5p	0.232
		hsa-let-7g-5p	0.238
		hsa-miR-191-5p	0.251
		hsa-miR-9-5p	0.264
		hsa-let-7f-5p	0.275
		hsa-miR-186-5p	0.286
		hsa-let-7a-5p	0.291
		hsa-miR-151a-3p	0.346
		hsa-miR-27a-3p	0.349
		hsa-miR-143-3p	0.350
		hsa-let-7c-5p	0.382
		hsa-miR-140-3p	0.391
		hsa-miR-101-3p	0.413
		hsa-miR-136-3p	0.444
		hsa-miR-382-5p	0.444
		hsa-miR-223-5p	0.444
		hsa-miR-627-5p	0.444
		hsa-miR-501-3p	0.444
		hsa-miR-181a-3p	0.444
		hsa-miR-222-3p	0.444
		hsa-miR-133a-3p	0.444
		hsa-miR-219a-2-3p	0.457
		hsa-miR-29a-3p	0.457
		hsa-miR-10a-5p	0.500
		hsa-let-7i-5p	0.500

Supplementary Table S2. The differential expressed miRNAs in recurrent KD (2ndKD-pre-IVIG) compared with healthyl control

microRNA upregulation	Fold change	microRNA downregulation	Fold change
hsa-miR-215-5p	4475.45	hsa-miR-106b-3p	0.002
hsa-miR-1304-3p	548.52	hsa-miR-342-3p	0.004
hsa-miR-31-5p	471.23	hsa-miR-4732-3p	0.005
hsa-miR-129-5p	413.89	hsa-miR-23a-3p	0.006
hsa-miR-34c-5p	349.06	hsa-miR-146a-5p	0.007
hsa-miR-497-5p	319.14	hsa-miR-223-3p	0.008
hsa-miR-99a-5p	261.80	hsa-miR-30e-5p	0.011
hsa-miR-125b-5p	243.36	hsa-miR-484	0.011
hsa-miR-299-3p	159.57	hsa-miR-6511a-3p	0.012
hsa-miR-500a-3p	149.60	hsa-miR-144-5p	0.013
hsa-miR-133a-3p	140.48	hsa-miR-122-5p	0.013
hsa-miR-222-3p	119.47	hsa-miR-421	0.014
hsa-miR-130b-3p	112.20	hsa-miR-128-3p	0.014
hsa-miR-3912-3p	99.73	hsa-miR-363-3p	0.015
hsa-miR-148a-5p	82.28	hsa-miR-3615	0.015
hsa-miR-29c-3p	69.81	hsa-miR-126-5p	0.016
hsa-miR-378a-3p	69.69	hsa-miR-98-5p	0.017
hsa-miR-375	57.35	hsa-miR-185-5p	0.017
hsa-miR-542-3p	52.36	hsa-miR-210-3p	0.018
hsa-miR-100-5p	48.01	hsa-miR-625-3p	0.019
hsa-miR-194-5p	38.97	hsa-miR-17-5p	0.023
hsa-miR-28-3p	36.81	hsa-miR-21-3p	0.025
hsa-miR-143-3p	32.32	hsa-miR-598-3p	0.026
hsa-miR-425-5p	27.47	hsa-miR-18a-3p	0.026
hsa-miR-192-5p	25.54	hsa-miR-664b-3p	0.028
hsa-miR-99b-5p	22.84	hsa-miR-877-5p	0.028
hsa-miR-27b-3p	17.72	hsa-miR-93-3p	0.030
hsa-miR-148a-3p	16.07	hsa-miR-139-5p	0.032
hsa-miR-1307-3p	15.49	hsa-miR-486-3p	0.032
hsa-miR-10a-5p	14.01	hsa-miR-20a-5p	0.034
hsa-let-7e-5p	13.00	hsa-miR-339-5p	0.034
hsa-miR-127-3p	11.46	hsa-miR-4742-3p	0.034
hsa-miR-125a-5p	10.32	hsa-miR-652-3p	0.034
hsa-miR-199a-3p	9.31	hsa-miR-335-5p	0.037
hsa-miR-199b-3p	9.31	hsa-miR-589-5p	0.037
hsa-miR-151a-3p	8.90	hsa-miR-3200-5p	0.037
hsa-miR-424-3p	8.11	hsa-miR-301a-3p	0.040
hsa-miR-150-3p	7.96	hsa-miR-4446-3p	0.040
hsa-miR-125b-2-3p	7.14	hsa-miR-6754-3p	0.040
hsa-miR-221-3p	7.05	hsa-miR-30c-5p	0.040
hsa-miR-26a-5p	7.04	hsa-miR-483-3p	0.049
hsa-miR-181b-5p	6.70	hsa-miR-126-3p	0.055
hsa-miR-26b-5p	6.10	hsa-miR-339-3p	0.055
hsa-miR-181a-5p	5.50	hsa-miR-32-5p	0.055
hsa-let-7a-5p	5.05	hsa-miR-636	0.055
hsa-miR-330-5p	4.99	hsa-miR-106a-5p	0.063
hsa-miR-22-3p	4.87	hsa-miR-548ad-5p	0.063
hsa-let-7f-5p	4.77	hsa-miR-548ae-5p	0.063
hsa-miR-145-5p	4.74	hsa-miR-548ay-5p	0.063
hsa-miR-30a-5p	4.69	hsa-miR-548d-5p	0.063
hsa-miR-184	4.42	hsa-miR-1180-3p	0.063
hsa-let-7i-5p	4.23	hsa-miR-4732-5p	0.063
hsa-miR-182-5p	4.09	hsa-miR-181c-5p	0.069
hsa-miR-532-5p	4.04	hsa-miR-22-5p	0.089
hsa-miR-181a-2-3p	3.97	hsa-miR-660-5p	0.089
hsa-miR-423-3p	3.84	hsa-let-7d-5p	0.105
hsa-miR-186-5p	3.70	hsa-miR-340-5p	0.108
hsa-miR-10b-5p	3.54	hsa-miR-338-3p	0.148
hsa-miR-21-5p	3.52	hsa-miR-204-5p	0.201
hsa-miR-24-3p	3.23	hsa-miR-93-5p	0.226
hsa-let-7c-5p	2.82	hsa-let-7b-5p	0.275
hsa-miR-423-5p	2.82	hsa-miR-451a	0.319
hsa-miR-30e-3p	2.62	hsa-miR-150-5p	0.348
hsa-miR-758-3p	2.49	hsa-miR-19b-3p	0.349
hsa-miR-149-5p	2.49	hsa-miR-19a-3p	0.411
hsa-miR-431-3p	2.49	hsa-miR-101-3p	0.421
hsa-miR-744-5p	2.49	hsa-miR-136-3p	0.444
hsa-miR-92b-3p	2.49	hsa-miR-382-5p	0.444
hsa-miR-130a-3p	2.48	hsa-miR-223-5p	0.444
hsa-miR-27a-3p	2.39	hsa-miR-627-5p	0.444
hsa-miR-409-3p	2.36	hsa-miR-501-3p	0.444
		hsa-let-7g-5p	0.500
		hsa-miR-9-5p	0.500

Supplementary Table S3. The differentially expressed miRNAs in patients with viral infection compared with healthy control

microRNA upregulation	Fold change	microRNA downregulation	Fold change
hsa-miR-345-5p	167.46	hsa-miR-19a-3p	0.002
hsa-miR-141-3p	160.18	hsa-let-7b-5p	0.003
hsa-miR-99a-5p	123.78	hsa-miR-532-5p	0.005
hsa-miR-222-3p	115.21	hsa-miR-27a-3p	0.005
hsa-miR-30a-3p	106.79	hsa-miR-93-5p	0.005
hsa-miR-10a-3p	97.08	hsa-miR-409-3p	0.007
hsa-miR-769-5p	89.80	hsa-miR-223-3p	0.008
hsa-miR-1304-3p	84.95	hsa-miR-145-5p	0.009
hsa-miR-96-5p	84.95	hsa-miR-183-5p	0.009
hsa-miR-3605-5p	70.38	hsa-miR-15b-5p	0.010
hsa-miR-100-5p	55.56	hsa-miR-130a-3p	0.011
hsa-miR-155-5p	48.54	hsa-miR-6511a-3p	0.012
hsa-miR-215-5p	43.69	hsa-miR-122-5p	0.013
hsa-miR-29a-3p	12.38	hsa-miR-151a-3p	0.013
hsa-miR-3614-5p	12.14	hsa-miR-421	0.014
hsa-miR-320c	9.71	hsa-miR-3615	0.015
hsa-miR-423-3p	7.56	hsa-miR-98-5p	0.016
hsa-miR-6087	7.28	hsa-miR-186-5p	0.017
hsa-miR-744-5p	4.85	hsa-miR-185-5p	0.017
hsa-miR-501-3p	4.31	hsa-miR-625-3p	0.019
hsa-miR-320b	4.25	hsa-miR-340-5p	0.021
hsa-miR-181c-5p	4.11	hsa-miR-126-3p	0.022
hsa-miR-28-3p	3.75	hsa-miR-17-5p	0.023
hsa-miR-9-5p	2.99	hsa-let-7d-5p	0.026
hsa-miR-660-5p	2.58	hsa-miR-598-3p	0.026
hsa-miR-29c-3p	2.43	hsa-miR-18a-3p	0.026
hsa-miR-199a-5p	2.43	hsa-miR-664b-3p	0.028
hsa-miR-338-5p	2.43	hsa-miR-93-3p	0.030
hsa-miR-410-3p	2.43	hsa-miR-139-5p	0.032
hsa-miR-138-5p	2.43	hsa-miR-486-3p	0.032
hsa-miR-10a-5p	2.33	hsa-miR-194-5p	0.034
hsa-miR-21-3p	2.15	hsa-miR-20a-5p	0.034
		hsa-miR-339-5p	0.034
		hsa-miR-4742-3p	0.034
		hsa-miR-652-3p	0.034
		hsa-miR-425-5p	0.037
		hsa-miR-424-3p	0.037
		hsa-miR-24-3p	0.037
		hsa-miR-335-5p	0.037
		hsa-miR-589-5p	0.037
		hsa-miR-3200-5p	0.037
		hsa-miR-301a-3p	0.040
		hsa-miR-4446-3p	0.040
		hsa-miR-6754-3p	0.040
		hsa-miR-30c-5p	0.040
		hsa-miR-150-5p	0.048
		hsa-miR-483-3p	0.049
		hsa-miR-339-3p	0.055
		hsa-miR-32-5p	0.055
		hsa-miR-636	0.055
		hsa-miR-106a-5p	0.063
		hsa-miR-548ad-5p	0.063
		hsa-miR-548ae-5p	0.063
		hsa-miR-548ay-5p	0.063
		hsa-miR-548d-5p	0.063
		hsa-miR-1180-3p	0.063
		hsa-miR-4732-5p	0.063
		hsa-miR-101-3p	0.077
		hsa-miR-19b-3p	0.078
		hsa-miR-181a-2-3p	0.080
		hsa-miR-378a-3p	0.083
		hsa-miR-106b-3p	0.085
		hsa-miR-150-3p	0.089
		hsa-miR-22-5p	0.089
		hsa-miR-125a-5p	0.090
		hsa-miR-125b-2-3p	0.098
		hsa-miR-204-5p	0.098
		hsa-miR-30d-5p	0.126
		hsa-miR-26a-5p	0.131
		hsa-let-7g-5p	0.137
		hsa-miR-451a	0.142
		hsa-miR-146b-5p	0.179
		hsa-miR-144-3p	0.191
		hsa-miR-191-5p	0.207
		hsa-miR-25-3p	0.209
		hsa-miR-181b-5p	0.259
		hsa-miR-182-5p	0.272
		hsa-miR-126-5p	0.273
		hsa-miR-140-3p	0.276
		hsa-miR-144-5p	0.285
		hsa-let-7i-5p	0.327
		hsa-let-7f-5p	0.341
		hsa-miR-338-3p	0.359
		hsa-let-7e-5p	0.389
		hsa-miR-133a-3p	0.444
		hsa-miR-181a-3p	0.444
		hsa-miR-136-3p	0.444
		hsa-miR-382-5p	0.444
		hsa-miR-223-5p	0.444
		hsa-miR-627-5p	0.444
		hsa-miR-92b-3p	0.461
		hsa-miR-4732-3p	0.484
		hsa-miR-92a-3p	0.493
		hsa-let-7a-5p	0.500
		hsa-miR-9-5p	0.500
